# Classification of First-Episode Psychosis with EEG Signals: ciSSA and Machine Learning Approach

**DOI:** 10.3390/biomedicines11123223

**Published:** 2023-12-05

**Authors:** Şerife Gengeç Benli

**Affiliations:** Department of Biomedical Engineering, Faculty of Engineering, Erciyes University, Kayseri 38280, Turkey; serifegengec@erciyes.edu.tr

**Keywords:** first-episode psychosis, electroencephalography, circulant spectrum analysis, machine learning

## Abstract

First-episode psychosis (FEP) typically marks the onset of severe psychiatric disorders and represents a critical period in the field of mental health. The early diagnosis of this condition is essential for timely intervention and improved clinical outcomes. In this study, the classification of FEP was investigated using the analysis of electroencephalography (EEG) signals and circulant spectrum analysis (ciSSA) sub-band signals. FEP poses a significant diagnostic challenge in the realm of mental health, and it is aimed at introducing a novel and effective approach for early diagnosis. To achieve this, the LASSO method was utilized to select the most significant features derived from entropy, frequency, and statistical-based characteristics obtained from ciSSA sub-band signals, as well as their hybrid combinations. Subsequently, a high-performance classification model has been developed using machine learning techniques, including ensemble, support vector machine (SVM), and artificial neural network (ANN) methods. The results of this study demonstrated that the hybrid features extracted from EEG signals’ ciSSA sub-bands, in combination with the SVM method, achieved a high level of performance, with an area under curve (AUC) of 0.9893, an accuracy of 96.23%, a sensitivity of 0.966, a specificity of 0.956, a precision of 0.9667, and an F1 score of 0.9666. This has revealed the effectiveness of the ciSSA-based method for classifying FEP from EEG signals.

## 1. Introduction

Psychotic disorders include a set of fundamental characteristics, including delusions, hallucinations, disordered thinking and speech, and negative symptoms, as outlined in the Diagnostic and Statistical Manual of Mental Disorders, Fifth Edition (DSM-5) [1]. The prognosis for individuals who undergo their initial episode of psychosis (FEP) shows considerable diversity. Whereas some patients may encounter only one psychotic episode, others may contend with recurring episodes or endure chronic symptoms associated with schizophrenia spectrum disorders. The use of early intervention strategies has the potential to significantly impact long-term results [2]. Diagnosing FEP involves a comprehensive psychiatric evaluation to assess symptoms, history, and any potential underlying medical or substance-related causes. Given the variability and complexity of psychotic disorders, an accurate diagnosis is essential for providing appropriate treatment and support to individuals experiencing FEP. Early intervention and treatment are crucial for improving outcomes and preventing potential relapses or chronicity of symptoms. In recent times, there has been a growing trend in utilizing computational methods to analyze neuroimaging data acquired from techniques like electroencephalography (EEG) and various magnetic resonance imaging modalities. These methods have become increasingly popular, as they offer valuable insights into the neurobiological underpinnings of psychiatric disorders due to the analysis of the whole brain.

There is an increasing interest in utilizing the features obtained from MR images for classification in distinguishing neuropsychiatric disorders [3,4,5,6,7]. In the study proposed by Squarcina et al., classification based on the measurement results of cortical thicknesses from different brain regions was performed, including 127 healthy controls and 127 individuals with FEP. When individuals were evaluated to classify, up to more than 80% for frontal and temporal areas was achieved in covariate multiple kernel learning, indicating that fronto-temporal cortical thickness could be used as a potential biomarker for classifying psychotic patients [8]. Faria et al. utilized different analysis methods to brain images from various MRI modalities belonging to 87 individuals of FEP and 62 healthy participants. These multiple approach methods have been more successful in distinguishing between healthy control and FEP subgroups using two-sample *t*-tests. Furthermore, the study highlights that the thalamus is significantly different in FEP [9]. In the study conducted by Nadal et al. involving 27 healthy individuals and 31 individuals with FEP, comparisons of brain volumes and nucleus accumbens volumes were performed based on structural MR images. In this study, where linear regression was applied, FEP exhibited an increased nucleus accumbens volume compared to controls [10].

When reviewing the studies in the literature, it is clear that there is a significant concentration of analyses focusing on artificial intelligence techniques involving EEG signals in healthy individuals, especially through multi-task-based approaches, for the development of brain–computer interface systems. On the other hand, there is a growing effort to develop patterns using multi-channel EEG data gathered from individuals, aiming to distinguish individuals with psychiatric disorders from healthy ones. There are only a few resting-state EEG studies aimed at finding patterns of EEG signals belonging to FEP. For instance, the study by Missonnier et al. was performed with eighteen healthy individuals and 15 individuals with FEP using 20-channel EEG signals. For these two groups, event-related gamma (35–45 Hz) and delta (0.5–4 Hz) oscillatory responses were evaluated in a visual n-back working memory task. Oscillation periodicity analyses were calculated to investigate the relationship between the psychiatric condition reflected by each frequency range and the working memory load. Statistical differences were obtained in sub-band oscillations between these two groups [11]. Focused on the delta frequency band of resting-state EEG signals, a study proposed by Renaldi et al. was conducted with 24 individuals with FEP and 24 healthy controls. Individuals with FEP had a significant increase in delta absolute power in both the frontal and posterior areas when compared to healthy controls. Furthermore, it was shown that the fluctuating influence of the delta frequency band had a crucial role in predicting the improvement of symptoms and functionality in those who underwent conventional therapy over the span of one year [12]. Lee et al. aimed to investigate the spatiotemporal neural correlations of cognitive function impairment in schizophrenia by focusing on resting-state EEG signals involving the default mode network regions. In this context, they conducted a study with 59 individuals with FEP and 50 healthy controls. In their study, they found that FEP patients exhibited a greater theta phase-gamma amplitude connectivity in the left posterior cingulate cortex compared to healthy controls [13]. In addition to the summarized FEP studies, researchers have focused on studies examining EEG frequency sub-bands in other psychiatric disorders [14,15].

Due to its nonlinear and non-stationary nature, EEG requires the application of certain signal processing techniques to reveal its intrinsic features. Specifically, examining the characteristics of EEG sub-bands obtained using techniques like empirical mode decompositions (EMD), variational mode decomposition (VMD), wavelet transform (WT), and singular spectrum analysis (SSA), or investigating the characteristic frequency sub-bands of EEG, are important approaches. Particularly, these approaches enhance success in resting-state or task-based EEG studies on psychiatric disorders or healthy controls. For instance, Aslan and Akin have proposed a deep learning study using two different datasets to differentiate between individuals with psychotic disorders and healthy controls. They employed the EMD approach to obtain four intrinsic functions, and the Hilbert spectrum images of the first intrinsic function were used as inputs for classification with the VGG16 model. They achieved accuracies of 98.2% and 96.02% for the first and second datasets, respectively, in the classification of these images, distinguishing between the two groups, thus demonstrating a remarkable ability to discern between the two cohorts [16]. In the study proposed by Khare and Bajaj, a robust VMD method was applied to multi-channel EEG signals in a cohort involving 49 individuals with schizophrenia and 32 healthy controls. In the classification using features extracted from intrinsic mode functions, an accuracy of 92.93% was achieved [17].

A novel approach has been proposed in the conducted study to achieve a high-performance classification of healthy individuals and those with first-episode psychosis (FEP) using EEG signals.

In pursuit of this objective, a new method called ciSSA was employed to uncover hidden patterns within the EEG signals and to reveal their dynamic behavior by decomposing them into sub-bands. Entropy, frequency, and statistical features were extracted from these sub-band signals, and machine learning techniques were applied to the obtained models to analyze their classification performance.

The contributions of this study to the literature are summarized as follows:

- EEG signals from the datasets used for FEP classification were analyzed for the first time in this study.
- The ciSSA method was applied for the first time within the scope of this study, specifically for the stated purpose, and its performance was analyzed.
- The classification performance of features obtained from both non-decomposed EEG signals and ciSSA-decomposed sub-band EEG signals was demonstrated.
- The novel entropy, statistical, and frequency features, combined with the ciSSA sub-bands of EEG signals for FEP classification, were analyzed for their performance.
- The classification performance of machine learning algorithms such as SVM, ANN, and ensemble methods in FEP classification using the new ciSSA-based model was examined.

## 2. Materials and Methods

In this study, new approaches have been proposed in terms of the methods and utilized dataset in the literature for the classification of FEP, resulting in the development of a high-performance classification model.

**Figure 1:**
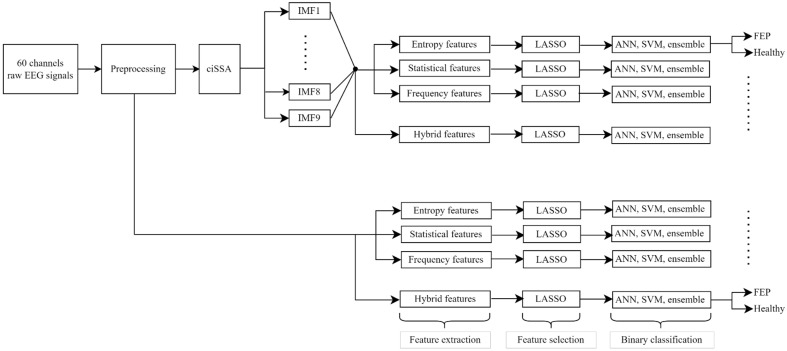
The entire framework of the proposed approach for the automated classification via EEG raw and ciSSA sub-band signals.

In this section, the dataset from which EEG signals were obtained, the preprocessing steps applied to the dataset, the sub-band decomposition procedures, the feature extraction and selection processes, and the classification algorithms are discussed. The framework of this study is presented in Figure 1.

### 2.1. Datasets

Two publicly available datasets [18,19] were used to classify FEP in this study. The study was conducted with two groups, a total of 78 individuals with FEP (23 females, 55 males) and 60 healthy controls (26 females, 34 males) across both datasets. The FEP group consisted of participants aged between approximately 12 and 36 (mean 22.83), whereas the control group ranged from 12 to 38 (mean 22.89). Forty-seven individuals with FEP and thirty healthy control individuals from the first dataset were included. From the second dataset, 31 individuals with FEP and 30 healthy controls were included. Demographic information about the individuals included in this study is presented in Supplementary Material S1. The EEG physiological signals of each individual were recorded during a resting state for a duration of 5 min with a 64-channel system using an Elekta Neuromag Vectorview system. The first 60 channels, containing only EEG signals, were used. Furthermore, the phenotype directory containing clinical assessment results and the data divided by type for all participants is detailed extensively in the relevant section of the datasets [18,19].

### 2.2. Preprocessing

**Figure 2:**
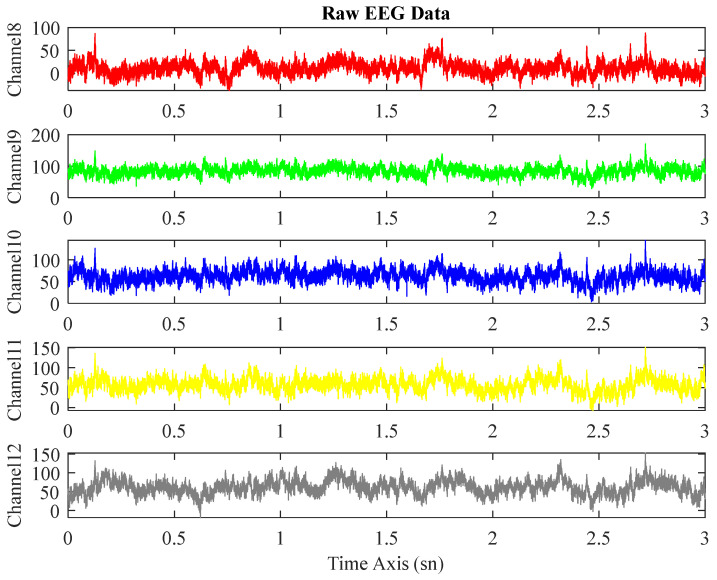
Multi-channel EEG signals before preprocessing.

**Figure 3:**
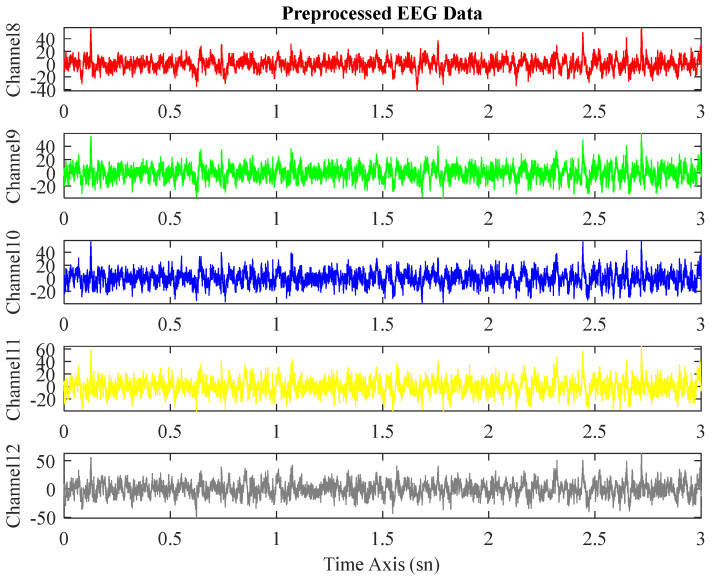
Multi-channel EEG signals after preprocessing.

This study uses the down-sampled data at 250 Hz, where the original sampling rate is 500 Hz. All included raw EEG records were preprocessed using the open-source software package EEGLAB [20] in MATLAB 2022a. These psychological signals were filtered with a 0.5–45 Hz bandpass filter. Furthermore, to remove noises from the records such as muscle, eye, heart, and line noise, among others, independent component analysis was performed. The first 3 min of the data consisting of EEG signals lasting for 5 min have been utilized in this study. The EEG signals before and after preprocessing are seen in Figure 2 and Figure 3.

### 2.3. Circulant Singular Spectrum Analysis

SSA, EMD, and RLMD, as nonparametric procedures, perform time series decomposition using distinct approaches and they come with advantages and disadvantages that can be chosen based on the characteristics of the data. The selection of which method to use depends on the nature of the data, the purpose, and the requirements. On the other hand, whereas SSA is commonly employed for general time series analysis, ciSSA is often preferred for the analysis of irregular time series such as biomedical data. The ciSSA method’s key benefit is that users may combine the extracted components according to their requirements, since the components are specified accurately by frequency. The circulant SSA approach comprises the embedding, decomposition, grouping, and reconstruction processes [21].

Embedding step: In the first step, the time series of each EEG channel is transformed into a multidimensional trajectory matrix by sliding it over a specific window length. This matrix organizes time series data in a space that represents different dimensions.

The trajectory matrix is defined according to Equation (1):

(1) $$X=\left[\begin{array}{cccc} x_{1} & x_{2} & \cdots & x_{N}\\ x_{2} & x_{3} & \cdots & x_{N+1}\\ \vdots & \vdots & \vdots & \vdots \\ x_{L} & x_{L+1} & \cdots & x_{T} \end{array}\right]\;$$

In this context, *T* represents the length of the time series *x_t_*, whereas *L* denotes the window length.

Decomposition step: The trajectory matrix is decomposed into components at different frequencies. In this step, matrix cross-correlations are used, leveraging second-order moments to obtain circulant matrix *Sc*. This allows the separation of components at different frequencies. Components of *Sc* are characterized by the following equations:

(2) $$s_{m}=\frac{1}{T-m}\overset{T-m}{\underset{t=1}{\sum }}x_{t}x_{t-m},\;\;\;m=0,\;1,\;\ldots ,\;L-1\;$$

The first-row components and eigenvectors of *S_C_* are obtained by Equations (3) and (4), respectively:

(3) $$\alpha_{m}=\frac{L-m}{L}s_{m}+\frac{m}{L}s_{L-m},m=0,\;1,\;\ldots L-1$$

(4) $$diag\left(\lambda_{1},\lambda_{2},\;\ldots \lambda_{L}\right)=U*S_{C}U$$

in which *U* represents the Fourier matrix. In order to get a more in-depth explanation, Refs. [22,23] should be consulted. The *k*-th eigenvalue (Equation (6)) and accompanying eigenvector are associated with the following frequencies, as given in Equation (5):

(5) $$f_{k}=\frac{k-1}{L}f_{s}$$

in which fs is the EEG signal sampling frequency.

(6) $$\lambda_{K}≅f\left(\frac{k-1}{L}\right)=\sum_{m=-\infty }^{\infty }s_{m}exp\left(i2\pi m\frac{k-1}{L}\right)\;\;$$

This allows us to define Equation (7), which states that the *X* trajectory matrix may be divided into *X_k_* elementary matrices:

(7) $$X=\sum_{k=1}^{L}x_{k}=\sum_{k=1}^{L}U_{k}U_{k}^{*}X\;\;\;\;$$

Diagonal Averaging: The decomposed components are further processed by performing diagonal averaging among those with similar frequencies. This process leads to obtaining higher-level components.

Grouping: Finally, the higher-level components are grouped, with each group representing a signal at a specific frequency.

The main goal of ciSSA is to separate complex and high-dimensional time series data into lower-dimensional components, revealing meaningful structures. This method can be particularly useful for analyzing time series data like brain activity, where the separation and analysis of signals at different frequencies are important.

**Figure 4:**
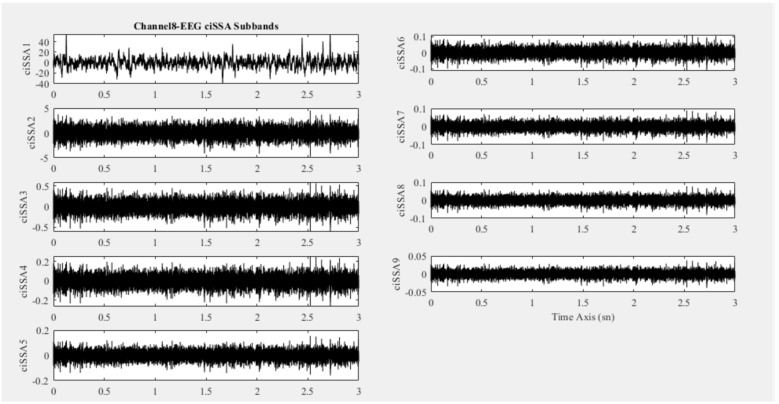
ciSSA sub-band signals belonging to channel 8 of an individual.

In this study, EEG signals were decomposed into sub-bands ranging from 4 to 15. Since the classification performance was optimal at 9, EEG signals were decomposed into 9 sub-bands using the ciSSA method. As an example, Figure 4 presents 9 ciSSA sub-bands of the eighth-channel EEG signal of an individual.

### 2.4. Feature Extraction

This work involves the extraction of three distinct kinds of feature datasets from EEG signals, including entropy, statistical, and frequency. These features are briefly mentioned below. Additionally, equations belonging to the features have been given in Supplementary Material S2.

#### 2.4.1. Entropy Features

Entropy is a method for quantifying the unpredictability of non-linear time series data. There are several derived variants of information entropy that are used in the processing of electroencephalography (EEG) data. Tsallis entropy [24,25], Tsallis entropy with different options, Shannon entropy [25], logenergy entropy [26], Renyi entropy [27], and Renyi entropy with different options were used to extract significant information from the EEG data. In total, 6 statistical features were included in the classification problem.

#### 2.4.2. Statistical Features

To perform tasks like feature extraction, classification, and pattern recognition, many different EEG applications rely on the data provided by statistical features. Thus, in this study, both basic and advanced statistical features have been used together in the classification problem. The arithmetic mean, median value, standard deviation, skewness, kurtosis, maximum, minimum, first difference, normalized first difference, second difference, normalized second difference, mean energy, mean teager energy, and log root sum of sequential variation were calculated for each channel signal. In addition to the other features, Hjorth parameters, including activity, mobility, and complexity, based on the variance of the derivatives of the EEG signal, have also been calculated. Thus, a total of 17 statistical features have been utilized.

#### 2.4.3. Frequency Features

Autoregressive (AR) models offer a wide range of applications in EEG signal processing, from estimating spectral properties and temporal dynamics to discriminating stationary signals. The AR model is very effective at representing and modeling the features and information contained in a signal. This model helps uncover patterns and relationships within brain activity [28]. In this study, an AR model was utilized to extract power spectral features from cortical EEG recordings. Furthermore, the order of the AR model was identified as 10, indicating that the AR-derived feature vector had 10 dimensions in any signal. In addition to the AR features, the power of the alpha, beta, delta, and theta bands and the ratio of band power alpha to theta were used as frequency features. In total, 15 features were included in this fiction.

### 2.5. Feature Selection

The high-dimensional nature of the EEG feature space makes feature selection inevitable. The advantage of feature selection in EEG signals lies in its ability to enhance the efficiency and effectiveness of analyzing and processing, e.g., increasing classification performance. In this study, the least absolute shrinkage and selection operator (LASSO) was performed for the purpose of feature selection. The LASSO, a statistical method introduced by Tibshirani [29], is used in regression analysis to perform parameter estimates and variable selection. LASSO may provide both an analytical solution and a low-variance estimate that is highly interpretable in the context of linear regression.

In this study, during the feature selection process using the LASSO method from the feature set obtained from the EEG signals, different lambda (*λ*) parameters were tested. Considering the computational load dependent on the number of features and the classification performance, the lambda value that provides the optimal solution has been determined as 0.005. The numbers of the features used in this study are given in the table below (Table 1).

### 2.6. Classification

In this study, after extracting the features and selecting the important ones, the binary classification of the EEG signals was performed using three supervised machine learning classifiers, such as SVM, ANN, and ensemble methods, via automated hyperparameter tuning. These classifiers are often used in several applications that include biological signals, such as EEG, as given in the [30,31] survey papers.

In the conducted study, the size of the dataset, the evaluation of desired outcomes, and factors such as the performance of algorithms in similar studies were taken into account when selecting these algorithms for the classification of the obtained features. Additionally, the ability of the chosen machine learning solution to effectively process the current data volume while maintaining accuracy and generalization capabilities has been assessed.

Cross-validation and testing on representative subsets of the used data were integral to my decision-making process. In summary, supporting the inclusion of ensemble models, SVM, and ANN while classifying them separately involves recognizing the individual strengths of each model, tailoring the evaluation process, and aiming for a comprehensive understanding of the research problem. This approach allows for a nuanced analysis, considering the diverse aspects captured by each model.

The next subsections provide concise explanations of the classifiers.

#### 2.6.1. Support Vector Machines

Support vector machines (SVMs) are a class of supervised machine learning algorithms used for classification and regression tasks. They are particularly effective for problems where the data are not linearly separable. SVMs work by finding a hyperplane that best separates different classes of data points in a high-dimensional space [32]. SVMs have been used to assist in diagnosing psychiatric disorders by analyzing EEG data for patterns indicative of conditions like attention deficit hyperactivity disorders [33], schizophrenia [34], and more.

#### 2.6.2. Artificial Neural Network

An artificial neural network (ANN) is a computational model that establishes a nonlinear relationship between input vectors and output vectors by the use of a network of linked neurons. Each node in the current layer is linked to every node in the subsequent and preceding layers. The output of a neuron is multiplied by the connecting weight and then sent forward to serve as an input, which is then processed by a nonlinear activation function for the neurons in the subsequent layer of the network. The use of nonlinear sigmoid activation functions is seen in both the hidden-layer neurons and the output-layer neurons inside a multilayer ANN [35]. ANN has been widely applied in various EEG-based studies [31,36,37].

#### 2.6.3. Ensemble Methods

Ensemble approaches involve the combination of multiple models aiming to address a common problem. The fundamental principle of this approach is that by bringing together these models, a more robust and accurate overall model can be created, thereby offering more reliable predictions and options compared to any individual model. The importance of aggregating the outcomes of many classifiers to reduce generalization errors has been emphasized [38,39]. Ensemble techniques have been shown to be particularly effective due to the existence of different inductive biases among various classifier types. These ensemble techniques have the potential to effectively harness the diversity present in a dataset to decrease variance error while minimizing bias error [40].

In this study, the classifier parameters for all classifiers were determined using the Bayesian optimization algorithm. The input feature vector was normalized to have a mean of 0 and a standard deviation of 1, and then applied to the classifier.

## 3. Results

This study was conducted using the signals of 60-channel EEG recordings taken from 60 healthy individuals and 78 individuals with first-episode psychosis. The preprocessed data were decomposed into nine sub-bands using the methods of ciSSA. In the feature extraction stage, features based on entropy, statistics, and the frequency of the original signal and its corresponding nine sub-bands obtained from each decomposition method were acquired for each channel.

In addition, among these obtained features, the most important ones have been identified for classification using the LASSO method in two approaches, i.e., both raw and ciSSA data.

The success of ciSSA sub-bands and the original signal in distinguishing FEP and healthy individuals has been examined using different approaches, including SVM, ensemble, and multilayer perception classifiers. The classification performance measures such as area under the curve (AUC), accuracy (Acc), sensitivity, specificity, and F1 score obtained from the binary classification have been evaluated. Table 2 and Table 3 show the mean classification performance as binary for all subjects through different feature types of datasets and hybrid datasets as raw signals and ciSSA sub-band signals, respectively. Upon examining these tables, the classification performance using features derived from the original data and ciSSA sub-band signals can be observed. During the classification study, the 10-fold cross-validation method was used to determine the training and testing data. Each classification process was repeated 10 times, and the given tables provide the mean performance achieved in the classification results.

**Figure 5:**
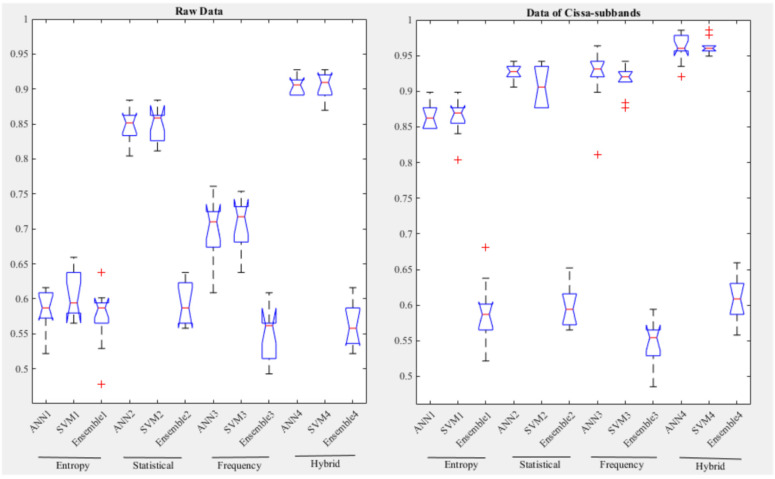
Box plots of classification accuracy obtained from various feature domains.

In this study, the classification performance results of 10 different trials using 10-fold cross validation are presented with box plots. This visual analysis demonstrates the stability of the proposed classification models and the consistency of the results, as seen in Figure 5. Box plots clearly illustrate the central tendencies, distributions, and potential outliers of the outcomes from various trials.

## 4. Discussion and Conclusions

The detection of first-episode psychosis is a crucial healthcare issue for both individuals and society, and accurate and early detection provides an opportunity for early intervention and treatment. Early treatment is important in reducing the severity of symptoms, slowing the progression of the illness, and improving the individual’s quality of life.

In the literature, many studies have been conducted to detect FEP using EEG signals. These studies have utilized various features extracted from EEG signals obtained from different datasets, including spectral power, phase-based and amplitude-based functional connectivity, macroscale network characteristics, density, power spectral density/spectral features, etc. [11,12,13,14,15]. Also, machine learning and statistical methods, either individually or together, have been utilized for classification purposes in the literature. Taking this into consideration, it would be appropriate to mention here that in this proposed study, only the machine learning approach has been applied. However, due to the limited number of studies incorporating a machine learning approach and the prevalence of literature reviews that predominantly focus on statistical evaluations, studies emphasizing statistical assessments have also been added to the comparative table.

An overview of the literature on studies using EEG signals for FEP detection can be seen in Table 4. These studies have shown classification performance ranging from 50.2% to 88.06%.

In this proposed study, a new approach was employed by decomposing EEG signals into ciSSA sub-band signals to obtain their different domain-specific features. This approach allowed for the extraction of important features from EEG signals for FEP detection from multiple perspectives. Using ensemble methods, as well as ANN and SVM machine learning techniques, this study presented a new method in the literature based on the feature and classifier combination that achieved the best classification performance.

As shown in Table 3, the classification results based on entropy features calculated from ciSSA sub-bands using ANN averaged 86.66% over 10 trials, whereas the statistical features and ANN yielded 92.53%, the frequency-based features and ANN achieved 92.02%, and all features with SVM resulted in a 96.23% classification success rate. As indicated in Table 2, in the approach that employed EEG signals without decomposing them into sub-bands and utilized entropy, statistical, frequency-based, and hybrid features with machine learning methods, a lower classification performance was obtained compared to the ciSSA feature machine learning approach.

Based on these results, a high-performance classification model for FEP detection from EEG signals was proposed, utilizing ciSSA sub-band signals and hybrid features consisting of entropy, statistical, and frequency features, along with an SVM model.

Upon analyzing literature reviews and the obtained results, it has been observed that significant advantages are achieved in this study compared to other studies utilizing statistical approaches, particularly in utilizing signal processing and machine learning models to achieve high accuracy and precision. Furthermore, the proposed combined model has demonstrated a substantial improvement in performance compared to other models employing machine learning approaches. In these regards, the suggested model provides a significant advantage by generating high-performance classification results. One of the most notable advantages of this study is the capability to classify the disease with high performance at its earliest stage. This contributes significantly to the accurate guidance of disease progression and treatment through early diagnosis. Additionally, in this study, 3-min EEG data were used. Revealing this system with recordings of such a short duration holds high potential for speed and performance in developing expert systems for this purpose. However, in the implementation stage of the model, the decomposition of data into sub-bands and the feature extraction from these sub-bands may introduce processing overhead. In this regard, the proposed model may have higher processing complexity compared to models directly analyzing the data.

In future studies, this method can be applied to the diagnosis of various neurological disorders, aiming to establish a ciSSA feature extraction machine learning model for the high-performance classification of neurological disorders.

## Figures and Tables

**Table 1:** Numbers of features used in the study.

EEG Signals Not Decomposed into Subbands				
	Entropy	Statictical	Frequency	Hybrid
Number of feature set	60 × 6 = 360	60 × 17 = 1020	60 × 15 = 900	60 × 38 = 2280
Number of selected features	70	104	106	128
**EEG Signals Secomposed into ciSSA Subbands**				
	**Entropy**	**Statistical**	**Frequency**	**Hybrid**
Number of feature set	60 × 6 × 9 = 3240	60 × 17 × 9 = 9180	60 × 15 × 9 = 8100	60 × 38 × 9 = 20,520
Number of selected features	164	153	141	181

**Table 2:** Classification results with various features obtained from the original EEG signals.

Features	Classification Methods	AUC	Accuracy	Sensitivity	Specificity	Precision	F-Score
Entropy	Ensemble	0.5477	0.5731	0.7333	0.365	0.606	0.6552
	SVM	0.6470	0.6050	0.6410	0.5583	0.6617	0.6429
	ANN	0.5893	0.5862	0.6346	0.5233	0.6407	0.6302
Statistical	Ensemble	0.6123	0.5920	0.7038	0.4466	0.6230	0.6604
	SVM	0.9074	0.8492	0.8448	0.855	0.883	0.8636
	ANN	0.9250	0.85	0.8410	0.8616	0.8878	0.8634
Frequency	Ensemble	0.5166	0.5471	0.8076	0.2083	0.5724	0.6605
	SVM	0.7501	0.7050	0.7128	0.695	0.7540	0.7324
	ANN	0.7549	0.6985	0.7217	0.6683	0.7385	0.7298
Hybrid	Ensemble	0.5747	0.5623	0.6589	0.4366	0.6034	0.6278
	SVM	0.9555	0.9036	0.9076	0.8983	0.9207	0.9140
	ANN	0.9549	0.9057	0.9038	0.9083	0.9280	0.9156

**Table 3:** Classification results with various features obtained from the ciSSA sub-bands of EEG signals.

Features	Classification Methods	AUC	Accuracy	Sensitivity	Specificity	Precision	F-Score
Entropy	Ensemble	0.5945	0.5862	0.6846	0.4583	0.6222	0.6513
	SVM	0.8941	0.8644	0.9025	0.815	0.8641	0.8827
	ANN	0.9043	0.8666	0.9115	0.8083	0.8609	0.8852
Statistical	Ensemble	0.6336	0.5971	0.7128	0.4466	0.6268	0.5439
	SVM	0.9454	0.9050	0.9307	0.8716	0.9039	0.9170
	ANN	0.9675	0.9253	0.9320	0.9166	0.9359	0.9337
Frequency	Ensemble	0.5391	0.5485	0.6615	0.4016	0.5925	0.6161
	SVM	0.9492	0.9159	0.9256	0.9033	0.9257	0.9255
	ANN	0.9592	0.9202	0.9179	0.9233	0.9389	0.9279
Hybrid	Ensemble	0.6327	0.6079	0.7269	0.4533	0.6340	0.6763
	SVM	0.9893	0.9623	0.9666	0.9566	0.9667	0.9666
	ANN	0.9880	0.9601	0.9564	0.965	0.9725	0.9643

**Table 4:** Summary of the classification performance of various scenarios with EEG signals from different datasets belonging to FEP.

Refs.	Dataset	Ch Size	FEP/Ctrl	Tasks/Duration	Signal Processing Methods/Features	Machine Learning Techniques/Statistics	Accuracy
[41]	Collected data	64 Ch	62/106	Resting state/45 min	Several features (60) of four fequency subbands (spectral power, phase-based and amplitude-based functional connectivity, and macroscale network characteristics were analyzed)	a random forest (RF) classifier/Mann–Whitney U test and RF regression were used for statistical analysis	50.2%
[42]	Collected data	19 Ch	29/25	Resting state	Microstate analysis/EEG microstate dynamics	ANOVAs	Between-group comparisons at baseline indicated significant differences
[43]	Collected data	19 Ch	17/30 mFEP/uFEP	Several tasks during 20 min	Microstate analysis/EEG microstates dynamics	ANOVA and *t*-test	Statistically significant differences were found
[44]	Collected data	64 Ch	20/33	Mismatch negativity (MMN) paradigm	Various measures from alpha, delta, and theta	k-means	Not presented
[45]	Collected data	64 Ch	26/17	Resting state/eyes open (EO) and eyes close (EC): EO (3 min), EC (3 min), EO (3 min), EC (3 min)	Power spectral density (PSD)/PSD vaues of EEG frequency subbands	channel-wise permutation-based statistics (paired Student’s *t*-tests and two-tailed were used; 1000 permutations)	There was no significant difference in EEG power between FEP and healthy controls in the following bands and conditions: AM/EC delta, theta, and higher alpha bands; PM/EC delta and alpha bands; AM/EO delta, theta, and alpha bands; PM/EO delta, theta, and lower alpha bands
[46]	Collected data	192 Ch	29/30	Resting state/10 min	Spectral power analysis/gamma spectral power	MANOVA and one-way ANOVA	The gamma spectral power in 31−50 Hz and 51–70 Hz frequency bands was found to be significantly higher in patients in most brain regions.
[47]	Collected data	64 Ch	16/11	After transcranial magnetic stimulation/na		not given detail	found no differences
[48]	Collected data	16 Ch	10/10	Emotional state (pleasant, unpleasant, neutral)	Wavelet coherence	Least-squares support-vector machine/ANOVA	83.89%, 86.39%, 88.06%, respectively/statistically significant differences were found
This proposed study	FEP1 [19] and FEP2 [18]	60 Ch	78/60	Resting state/3 min	ciSSA/Entropy, statistical, and frequency features	SVM, Ensemble classifier, Multi-layer perceptron-ANN	Given binary classifier results at Table 2 and Table 3

Ch: channels, Ctrl: healthy controls.

## Data Availability

The datasets used are publicly available at the given links below: https://openneuro.org/datasets/ds003944/versions/1.0.1 (accessed on 10 February 2023) and https://openneuro.org/datasets/ds003947/versions/1.0.1 (accessed on 10 February 2023).

## Supplementary Materials

The following supporting information can be downloaded at: https://www.mdpi.com/article/10.3390/biomedicines11123223/s1, Supplementary Materials S1: Demographic Informations of Participants; Supplementary Materials S2: Features Descriptions.
