# Supplementary material for: Classification of First-Episode Psychosis with EEG Signals: ciSSA and Machine Learning Approach
Source: Biomedicines. 2023 Dec 5;11(12):3223. doi: 10.3390/biomedicines11123223 (PMC10741114; doi:10.3390/biomedicines11123223)
Supplement: Supplementary file 1 [file biomedicines-11-03223-s001.zip › Supplementary Materials S2.pdf]

Supplementary Table S1. *Entropy features extracted from EEG signals*

Features	***Definitions***
Tsallis entropy [24]	$S_q = \frac{1}{q-1}\left(1 - \sum_{i=1}^{W} p_i^q\right)$
Shannon entropy [26]	$H(X) = \sum_{i=1}^{n} P(x_i) \,.\, log_b\, P(x_i)$
Logenergy entropy [26]	$E(k) = log\ (\lvert X(k)\rvert^2 + \epsilon)$
Renyi entropy [27]	$H_\alpha(X) = \frac{1}{\alpha-1} . log\left(\sum_{i=1}^{n} p_i^\alpha\right)$

Supplementary Table S2. *Statistical features extracted from EEG signals*

Features	***Definitions***
Arithmetic mean (μ)	$\mu = \frac{\sum_{i=1}^{N} x_i}{N}$
Median value	$Median = X_{\frac{N+1}{2}}$
Standart deviation	$\sigma = \sqrt{\frac{\sum_{i=1}^{n}(x_i - \mu)^2}{n}}$
Skewness	$Skewness = \frac{\sum_{i=1}^{n}(x_i - \mu)^3/n}{\left(\frac{\sum_{i=1}^{n}(x_i - \mu)^2/n}{n}\right)^3}$
Kurtosis	$Kurtosis = \frac{\sum_{i=1}^{n}(x_i - \mu)^4/n}{\left(\frac{\sum_{i=1}^{n}(x_i - \mu)^2/n}{n}\right)^2}$
Maximum	$Maximum = max\ (X)$
Minimum	$Minimum = min\ (X)$
First difference	$First\ Difference(X_i) = X_{i+1} - X_i$
Normalized first difference	$Normalized\ F.D.(X_i) = \frac{X_{i+1} - X_i}{X_i}$
Second difference	$S.Difference(X_i) = X_{i+2} - 2X_{i+1} + X_i$
Normalized second difference	$N.S.Difference(X_i) = \frac{X_{i+2} - 2X_{i+1} + X_i}{X_i}$
Mean energy	$E = \frac{1}{N}\sum_{n=0}^{N-1} \lvert x(n)\rvert^2$
Mean teager energy	$TE(x[n]) = x[n].x[n] - x[n-1].x[n+1]$
log root sum of sequential variation	$L.R.S.S.V = log\sqrt{\sum_{i=1}^{N-1}(x_{i+1} - x_i)^2}$
Hjorth activity	$Hjorth\ Act. = var(X(t))$

Hjorth mobility	$Hjorth\ Mob. = \sqrt{\frac{Var(X'(t))}{Var(X(t))}}$
Hjorth complexity	$Hjorth\ Comp. = \frac{mobility(X'(t))}{mobility(X(t))}$

Supplementary Table S3. *Frequency features extracted from EEG signals*

Features	***Definitions***
Power of alpha	$Power\ of\ Alpha = \int_{Alpha\ Band} P(f)df$
Power of beta	$Power\ of\ Beta = \int_{Beta\ Band} P(f)df$
Power of delta	$Power\ of\ Delta = \int_{Delta\ Band} P(f)df$
Power of theta	$Power\ of\ Theta = \int_{Theta\ Band} P(f)df$
Ratio of band power alpha to theta	$Ratio = \frac{Power\ of\ Alpha}{Power\ of\ Theta}$
AR features	$x(t) = \sum_{i=1}^{p} \emptyset_i\, x(t-i) + \epsilon(t)$
